# Potential digenic inheritance of familial hypertrophic cardiomyopathy identified by whole‐exome sequencing

**DOI:** 10.1002/mgg3.1150

**Published:** 2020-01-20

**Authors:** Ming‐Bao Ren, Xiao‐Rui Chai, Lin Li, Xin Wang, Chenghong Yin

**Affiliations:** ^1^ Department of Obstetrics Beijing Obstetrics and Gynecology Hospital Capital Medical University Beijing China; ^2^ Clinical Laboratory Medicine Center Fuwai Hospital Chinese Academy of Medical Sciences and Peking Union Medical College Beijing China; ^3^ Central Laboratory Beijing Obstetrics and Gynecology Hospital Capital Medical University Beijing China

**Keywords:** hypertrophic cardiomyopathy, *MYH7*, *TNNI3*, whole‐exome sequencing

## Abstract

**Background:**

The aim of this study was to identify the genetic causes of patients with hypertrophic cardiomyopathy (HCM) within a family. Most of the previous studies found point mutations as the genetic causes for HCM, whole‐gene deletion was rarely reported.

**Methods:**

Although, clinical genetic testing has been widely used for identifying variants in HCM patients, structural variations are understudied, partly owing to the inadequacy of the available methodology. In the present study, whole‐exome sequencing (WES) and Sanger sequencing validation was used to identify the genetic causes in patients with familial HCM.

**Results:**

A genomic deletion in Chromosome 19 containing the whole of troponin I3 gene (*TNNI3*), and the p.Ile736Thr variant in the myosin heavy chain 7 gene (*MYH7*) were identified in two patients with familial HCM by WES. The p.Ile736Thr variant is further validated by Sanger sequencing and is predicted as a pathogenic variant by in silico analysis.

**Conclusion:**

We added the notion that not only p.Ile736Thr variant of *MYH7*, but also *TNNI3* deletion might potentially contribute to HCM pathogenesis. Our study also suggested WES was a powerful tool to identify the genetic variants causing HCM.

## Graphical abstract

p.Ile736Thr variant of MYH7 is associated with HCM.

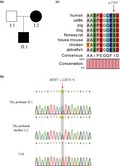

## 1 INTRODUCTION

Hypertrophic cardiomyopathy (HCM) is a genetic cardiac disease characterized by left ventricular hypertrophy (Gersh et al., 2011). HCM is diagnosed in patients with left ventricular wall thickness of more than 15 mm. HCM is a common disorder and affects more than 0.2% of the general population (Semsarian, Ingles, Maron, & Maron, 2015). Genetic factors contribute to approximately 50% of all cases of HCM, which are thought to be inherited in an autosomal dominant manner. HCM is usually caused by variants in sarcomere‐related genes, including *MYBPC3*, *MYH7*, *TNNT2*, *TNNI3*, *TPM1*, *ACTC1*, *MYL2*, *MYL3,* and other genes (Ingles et al., 2019; Liew, Vassiliou, Cooper, & Raphael, 2017). Most HCM patients are found to carry one pathogenic allele; however, a minority harbor more than one variant in one gene or two/three distinct genes. This may result in a more severe clinical phenotype with a higher incidence of heart failure or sudden death (Ingles et al., 2005; Maron, Maron, & Semsarian, 2012; Zheng et al., 2016). Recently, the oligogenic inheritance of congenital heart disease has been proved by the experimental model (Gifford et al., 2019). Although a panel of 7–10 HCM‐associated genes have been used for HCM patient screening (Das, Ingles, Bagnall, & Semsarian, 2014), structural variations cannot be easily detected by Sanger sequencing or targeted sequence capture methods. Thus, it is likely that important genetic variants in certain HCM patients might be overlooked.

In this study, we recruited two patients from a family with history of HCM. Whole‐exome sequencing (WES) and pedigree analyses revealed the existence of two variants in each patient. One identified variant included the whole‐gene deletion of troponin I3 (*TNNI3)*, which traditional Sanger sequencing might be unable to detect. Therefore, we conclude that WES is likely a powerful tool to identify both the point mutations and larger deletions/insertions involved in HCM.

## 2 MATERIALS AND METHODS

### 2.1 Patients

**Figure 1:**
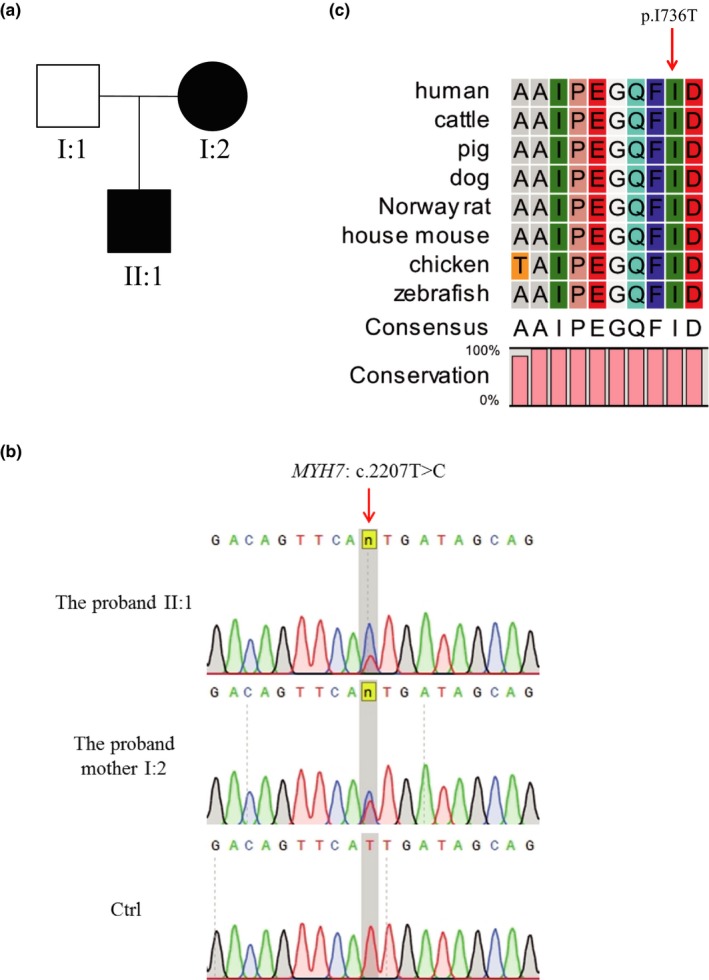
Pedigree analysis and sequence alignment analysis of MYH7 protein. (a) The family tree shows two patients with HCM. The black cycle and black square indicate the patients. (b) Sanger sequencing validated the *MYH7* variant in this family. Sanger sequencing confirmed the heterozygous *MYH7* variant in the two patients. The red arrow points to the mutational site (c.2207T>C). (c) Sequence alignment of MYH7 protein in different species. The red arrow indicates the p.Ile736 site, which is 100% conserved in different species

A Chinese family with two diagnosed HCM members was recruited for this study. The proband (Figure 1, II:1) and his mother (Figure 1, I:2) were diagnosed with HCM based on the HCM diagnostic criteria (Gersh et al., 2011). The thickness of the left ventricular wall of the proband's heart was 19.2 mm. The proband was 36‐year‐old male, and his mother was 62‐year‐old. The proband has a history of syncope after sports (playing basketball), and has no other special symptoms in daily life.

Detailed ultrasound description of the proband's heart is described below: the left ventricle diameter was in the normal range and the interventricular septum was thickened with the thickest part located in the anterior septum measuring approximately 19.2 mm. There were no obvious abnormalities in the motion of left ventricular wall. There was no obvious abnormality in the overall contraction or synergy, and the motion score of the left ventricular wall was 16 points. The size of the left atrial was normal with good cavity echo; the size of right heart was normal, no obvious abnormalities were observed in wall motion; no obvious interrupted echo was detected in the atrial septum, CDFI showed small blood flow from left to right with an approximate width of flow beam of 2.5 mm. There was no obvious interrupted echo in the interventricular septum, and no obvious blood flow was detected by Doppler. There was no obvious abnormality in the mitral valve echo. Mitral regurgitation (MR) was detected by CDFI during systole; no obvious abnormality of the aortic valve echo was observed and the opening function was normal, similar to the reflux and tricuspid in diastolic CDFI. CDFI trachea probe showed a small amount of return flow, which led to inaccurately determining the pressure difference; the pulmonary valve echo was normal with normal opening, the blood flow velocity of the systolic valve was not fast and no abnormal blood flow was found during diastole. The morphology of the ascending aorta and the inner diameter of the pulmonary artery were normal and no abnormal blood flow was detected. There was no apparent abnormality in pericardial echo. Therefore, the ultrasound results were suggestive of hypertrophic cardiomyopathy, normal left ventricular function, and patent foramen ovale.

This work has been approved by the Ethics Committee of Beijing Obstetrics and Gynecology Hospital. Written consents were obtained from all participants before samples were collected. Five milliliters of peripheral blood were collected from each patient.

### 2.2 Whole‐exome sequencing analysis

Whole‐exome sequencing (WES) was performed on DNA samples from the proband and his mother. WES was performed by a commercial sequencing service (Annoroad Gene Technology). All exons were captured and enriched using SureSelect Human All Exon V6 kit (Agilent Technologies). After the DNA libraries were prepared, they were sequenced with the HiSeq^TM^ PE150 system. Clean reads were obtained by removing the contaminant reads from the raw reads. Then, clean reads were aligned to the UCSC hg19 reference genome by Burrows‐Wheeler Aligner. High‐quality BAM files were generated by using Samtools and Picard, and variants were called from BAM files by GATK. Single‐nucleotide polymorphisms (SNPs) and insertion‐deletions (InDels) were annotated by ANNOVAR. Structural variations (SVs) were firstly detected by DELLY2 software and then were annotated by ANNOVAR. SVs referred to five different genetic variation types including deletions, duplications, insertions, inversions, and transversions. The data that support the findings of this study are available on request from the corresponding author. The data are not publicly available due to privacy or ethical restrictions.

## 3 RESULTS

DNA samples from both the proband (II:1) and his mother (I:2) were subjected to WES analysis. Pedigree analysis suggested a dominant mode of inheritance (Figure 1a). Previous studies have shown that mutations in 57 genes can cause or be associated with HCM, including 8 definitive genes (*MYBPC3*, *MYH7*, *TNNT2*, *TNNI3*, *TPM1*, *ACTC1*, *MYL2*, and *MYL3*), 3 moderate evidenced genes (*CSRP3*, *TNNC1*, and *JPH2*) and other limited or no evidenced genes (such as *TTN*, *KLF10*, *MYPN*, *ANKRD1*, *MYLK2*, *MYOZ2*, *NEXN*, *VCL*, *TRIM63*, *RYR2*, *MYH6*, *OBSCN*, *PDLIM3*, *TCAP*, *MYOM1*, and *CALR3*) (Das et al., 2014; Green et al., 2013; Ingles et al., 2019; Liew et al., 2017). Therefore, we focused on the genetic variations (SNPs, InDels, and SVs) occurring in any of the above‐mentioned genes. Moreover, we considered only the genetic variations that existed in both patients.

Based on the above‐mentioned strategy for variation analysis, a known pathogenic heterozygous missense variant in the myosin heavy chain 7 gene, *MYH7* (NM_000257:exon20:c.2207T>C:p.Ile736Thr) was identified in both patients and was further validated by Sanger sequencing (Figure 1b). The p.Ile736Thr variant was very conserved from human to zebrafish (Figure 1c). The allele frequency of c.2207T>C in *MYH7* was 0 in gnomAD, ExAC, 1,000 Genomes and ESP6500 exome or genome sequencing databases (Table 1). In silico analysis by Polyphen‐2, SIFT, PROVEAN, MutationTaster, SNPs&GO and FATHMM‐MKL suggested that p.Ile736Thr variant was a disease‐causing variant (Table 1). This variant was also interpreted as a pathogenic variant by ClinVar (https://www.ncbi.nlm.nih.gov/clinvar/variation/164342/).

**Table 1:** In silico analysis of MYH7 variant

Variants	Amino acid change	Polyphen‐2^a^	SIFT^b^	PROVEAN^c^	Mutation Taster^d^	SNPs&GO^e^	FATHMMMKL^f^	gnomAD^g^	ExAC^h^	1000 Genomes^i^	ESP6500^j^
c.2207T>C	p.Ile736Thr	Probably damaging (0.999)	Damaging (0.001)	Deleterious (‐3.54)	Disease causing (0.9999)	Disease (0.743)	Damaging (0.986)	0	0	0	0

a Polyphen‐2. Prediction Scores range from 0 to 1 with high scores indicating probably or possibly damaging.

b SIFT, i.e., Sorting Intolerant From Tolerant. Scores vary between 0 and 1. Variants with scores close or equal to 0 are predicted to be damaging.

c PROVEAN. Variants with scores lower than ‐2.5 (cutoff) are predicted to be deleterious.

d Mutation Taster. The probability value is the probability of the prediction, i.e., a value close to 1 indicates a high 'security' of the prediction.

e SNPs&GO. Probability: Disease probability (if >0.5 mutation is predicted Disease).

f FATHMM‐MKL. Values above 0.5 are predicted to be deleterious, while those below 0.5 are predicted to be neutral or benign.

g Frequency of variation in total of gnomAD database.

h Frequency of variation in total of ExAC database.

i Frequency of variation in 1000 Genomes database.

j Frequency of variation in ESP6500 database.

**Figure 2:**
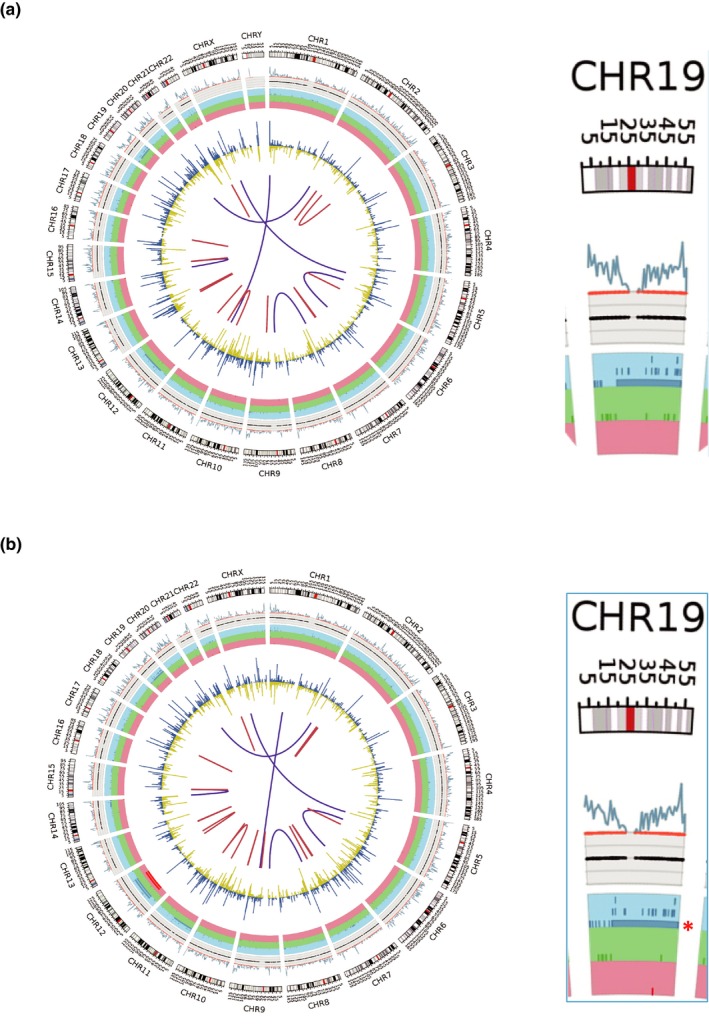
A genomic deletion in Chromosome 19 was detected in two HCM patients. (a) Circos plot containing a global overview of all genetic variations for Patient II:1. The dark blue line indicates deletion structural variations. The red asterisk indicates the 43 Mb deletion detected. The panel on the right is the enlarged representation of the red rectangle. (b) Circos plot for Patient I:2

Besides SNPs, SVs were also examined and analyzed. A heterozygous deletion in chromosome 19 was detected in both patients (Figure 2a,b). This large region in chromosome 19 contains the whole‐gene of *TNNI3*. Therefore, both HCM patients harbored a *MYH7* variant and a *TNNI3* whole‐gene deletion.

## 4 DISCUSSION

In this study we detected two variants in all HCM patients studied and having the same pedigree. One variant was the whole gene deletion of *TNNI3*. The *TNNI3* is located on chromosome 19q13.42; its encoded protein is expressed in cardiac muscle tissues and is one of three subunits forming the troponin complex. *TNNI3* is the seventh HCM‐associated gene to be discovered (Kimura et al., 1997), and contributes to 4%–8% of all HCM patients (Liew et al., 2017). The *TNNI3* is highly constrained and intolerant to loss‐of‐function variations (Marian & Braunwald, 2017). To date, about 30 different *TNNI3* variants have been linked to HCM (Zhao et al., 2015), however, as far as we are aware no whole *TNNI3* deletion has been associated with HCM. Therefore, our study is the first to report a heterozygous *TNNI3* genomic locus deletion in HCM patients. Previous studies have identified *TNNI3* missense variants by Sanger sequencing or targeted next‐generation sequencing for specific genes, thus increasing the likelihood of larger genomic deletions or insertions to be overlooked. Whole‐exome sequencing technology can detect both SNPs and structural variations. Thus, from the cases in our study we recommend that WES is a more efficient tool for the identification of genetic variations in the patients with HCM.

The second variant we identified in both HCM patients was located in the *MYH7* (NM_000257:exon20:c.2207T>C:p.Ile736Thr). Approximately 30% of all HCM cases are associated with variants in the *MYH7* (Richard et al., 2003). The p.Ile736Thr *MYH7* variant is a known pathogenic allele and has been reported by several studies (Barriales‐Villa et al., 2010; Erdmann et al., 2003; Perrot et al., 2005). Nevertheless, the p.Ile736Thr variant has also been considered a benign variant, due to its association with close to normal life expectancy (Tripathi et al., 2011).

Recently, Gifford et al., identified three missense variants in *MKL2* (Gln670His), *MYH7* (Leu387Phe), and *NKX2‐5* (Ala119Ser) in three offspring with childhood‐onset cardiomyopathy (Gifford et al., 2019). By using CRISPR‐Cas9 technology the authors generated mice encoding the orthologous variants and found that triple‐compound heterozygous mice recapitulated the human disease phenotype (Gifford et al., 2019). In our study, we found two distinct variants, *MYH7* missense variant (p.Ile736Thr) and *TNNI3* deletion. So in order to confirm the digenic inheritance pattern, further functional studies such as using CRISPR‐Cas9 technology to generate the compound heterozygous mice are needed.

Even if our study found that HCM patients might potentially harbor *TNNI3* deletion, we still need to realize that, given that these two patients have a known pathogenic *MYH7* variant, the clinical consequence of the *TNNI3* deletion is uncertain, in the absence of further family members that may have only one of the two variants. Therefore, we did not rule out that *TNNI3* deletion might be a nonpathogenic factor.

In summary, our study identified the first HCM case with whole *TNNI3* deletion, and we further provide evidence that WES is a powerful tool to comprehensively analyze genetic variants in HCM patients. Therefore, our study added the notion that digenic inheritance may contribute to HCM pathogenesis.

## CONFLICT OF INTEREST

The authors declare no conflict of interest.

## AUTHOR CONTRIBUTIONS

MBR, XRC, and LL were involved in data analysis of the whole‐exome sequencing. MBR, XW, and XRC were involved in clinical data collection. LL and CY were involved in manuscript preparation.
